# High-resolution crystal structure and biochemical characterization of a GH11 endoxylanase from *Nectria haematococca*

**DOI:** 10.1038/s41598-020-72644-w

**Published:** 2020-09-24

**Authors:** Hina Andaleeb, Najeeb Ullah, Sven Falke, Markus Perbandt, Hévila Brognaro, Christian Betzel

**Affiliations:** 1 Institute of Biochemistry and Molecular Biology, Laboratory for Structural Biology of Infection and Inflammation, University of Hamburg, c/o DESY, Build. 22a. Notkestr. 85, 22603 Hamburg, Germany; 2 Department of Biochemistry, Bahauddin Zakariya University, Multan, 60800 Punjab Pakistan; 3 The Hamburg Centre for Ultrafast Imaging (CUI), Luruper Chaussee 149, 22761 Hamburg, Germany

**Keywords:** Biochemistry, Biotechnology, Structural biology

## Abstract

Enzymatic degradation of vegetal biomass offers versatile procedures to improve the production of alternative fuels and other biomass-based products. Here we present the three-dimensional structure of a xylanase from *Nectria haematococca* (NhGH11) at 1.0 Å resolution and its functional properties. The atomic resolution structure provides details and insights about the complex hydrogen bonding network of the active site region and allowed a detailed comparison with homologous structures. Complementary biochemical studies showed that the xylanase can catalyze the hydrolysis of complex xylan into simple xylose aldopentose subunits of different lengths. NhGH11 can catalyze the efficient breakdown of beechwood xylan, xylan polysaccharide, and wheat arabinoxylan with turnover numbers of 1730.6 ± 318.1 min^−1^, 1648.2 ± 249.3 min^−1^ and 2410.8 ± 517.5 min^−1^ respectively. NhGH11 showed maximum catalytic activity at pH 6.0 and 45 °C. The mesophilic character of NhGH11 can be explained by distinct structural features in comparison to thermophilic GH11 enzymes, including the number of hydrogen bonds, side chain interactions and number of buried water molecules. The enzymatic activity of NhGH11 is not very sensitive to metal ions and chemical reagents that are typically present in associated industrial production processes. The data we present highlights the potential of NhGH11 to be applied in industrial biomass degradation processes.

## Introduction

Biofuels are an alternative to fossil fuels. Till now, increasing demand for fuels triggered substantial scientific research activities focused to optimize biofuel production. Agricultural wastes, especially sugar cane bagasse, can be treated with carbohydrate degrading enzymes to make them applicable for biofuel production^1,2^. In this context, research activities in the field of biofuel production are aiming at the potential of lignocellulosic material and carbohydrate degrading enzymes for bioethanol production. Carbohydrate degrading enzymes are also essentially used in the production pipelines of paper and food industries. In combination with other carbohydrate degrading enzymes, xylanases are utilized in bio-refineries, chemical and pharmaceutical industries, processing of fruit juices and production of prebiotics^3–8^.

In nature, either a single organism or a symbiotic relationship with bacteria and fungi triggers the degradation of lignocellulosic biomass using a variety of enzymes^9,10^. Glycosyl hydrolases (GHs) are key enzymes hydrolyzing lignocellulose material, and releasing saccharides as product that can for example be used in bio-refineries as carbon source to generate a variety of products^11^.

*Nectria haematococca* is a teleomorphic state of *Fusarium solani* which is a pathogenic fungus for several agricultural plant species^12^. It encodes 67 cellulases from different classes and 6 xylanases that belong to GH10 and GH11 families (www.uniprot.org). Endo-β-1,4-xylanases (EC 3.2.1.8) of the GH11 family are capable of hydrolyzing β-1,4 glycosidic bonds between two D-xylopyranosyl residues linked in xylan polymers. Xylan is one of the most abundant polysaccharide and covers 33% of the hemicellulose. Naturally, xylan is variously branched with sugars such as arabinose, xylose, and galactose or organic acids such as acetic acid, ferulic acid and glucuronic acid^13^.

Two highly conserved glutamates at the active site of GH11 xylanases trigger the hydrolysis of β-1,4 xylosidic linkages in xylan. In the first step of catalysis, the acid/base residue E180 (numbering to NhGH11) acts as an acid and protonates the xylan, while the other catalytic residue E89 acts as a nucleophile that provokes the departure of the leaving group to form a α-glycosyl enzyme intermediate. In the second step, the acid/base residue E180 act as a base and deprotonates the neighboring nucleophilic water, which attacks the anomeric carbon of the α-glycosyl enzyme intermediate and allows the product formation with β-configuration^14^.

Within the large pool of fungal xylanases NhGH11 has been found to be homologous to β-1,4-xylanases that have already industrial applications^15,16^. Referring to the carbohydrates active enzymes database (CAZy), the *N. haematococca* genome revealed several putative and till now structurally uncharacterized xylanases, including three from the family GH11 (CAZy; http://www.cazy.org). NhGH11 was therefore selected to analyze the structure, enzymatic activity and specificity to explore its potential to be utilized in industrial applications.

## Results and discussion

### Protein expression and purification

The production of highly active enzymes in bulk amounts required for industrial applications need to be economically optimized^17^. Therefore, the selection of the expression host essentially determines the production cost of commercial enzymes. Although *E. coli* is typically a preferable host, however till now many important xylanases could not be produced by *E. coli* in soluble form, mainly due to the repetition of rare codons or the absence of post-translational glycosylation^18,19^. To overcome this and to obtain NhGH11 in amounts required for crystallization experiments and biochemical investigations, recombinant gene expression was performed by using a maltose-binding-protein (MBP) as fusion construct and to improve solubility^20^. The use of MBP increased the protein expression efficiency substantially and was further applied as an affinity tag for purification. NhGH11 remained soluble after cleaving the MBP tag. Data about the purification of the fusion protein (MBP-NhGH11), cleavage of the tag, size exclusion chromatography and dynamic light scattering experiments are summarized in the Supplementary material (S1).

### Overall crystal structure

**Figure 1:**
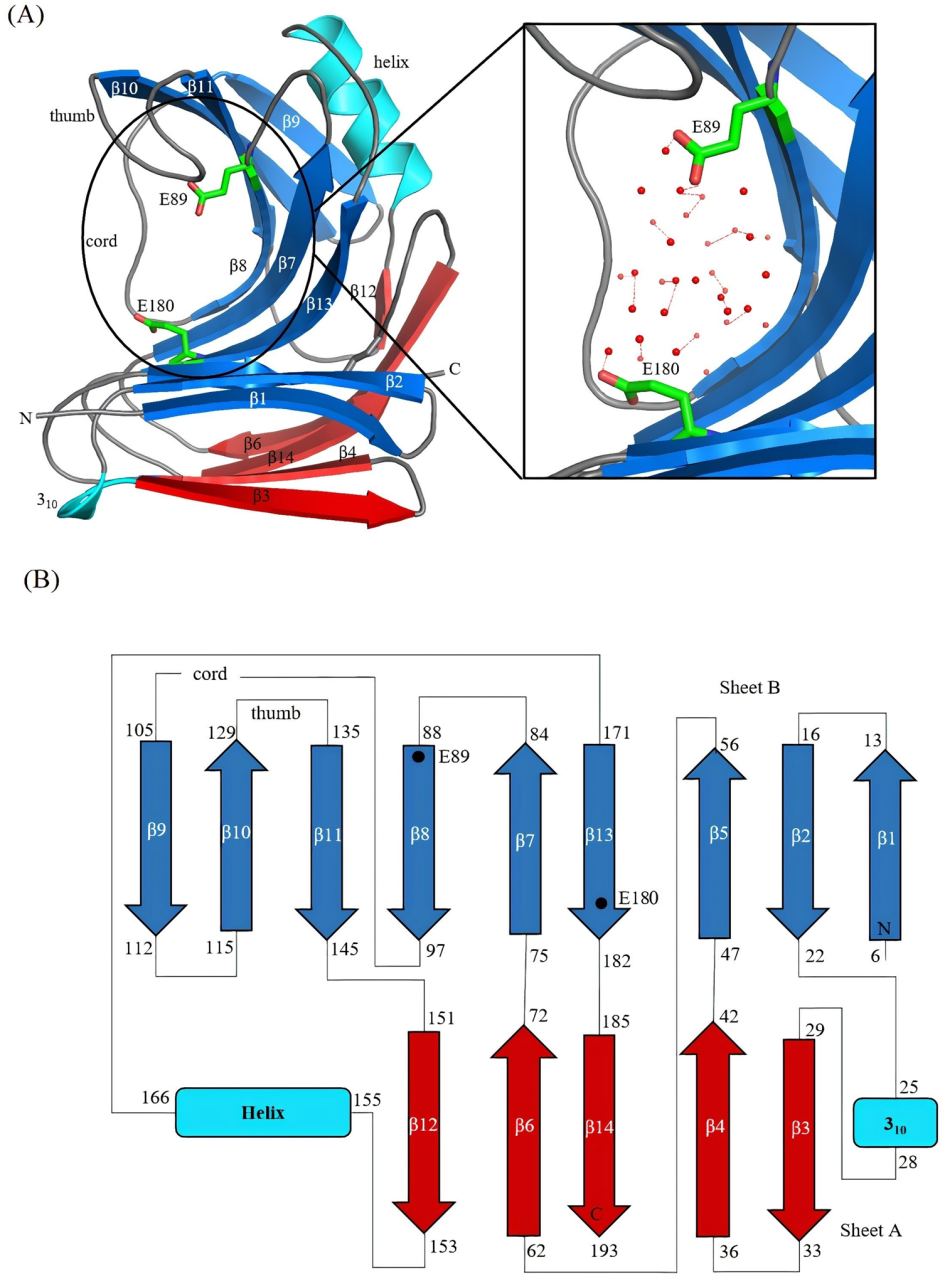
(**A**) Cartoon plot of NhGH11 with catalytic active residues are shown in sticks. The structure has a jelly-roll fold characteristic for family GH11 enzymes. The linker region, named cord, and the thumb are indicated. A zoom of the active site region is shown (right figure), with catalytic residues surrounded by solvent molecules in the active site cavity. (**B**) Topology diagram for NhGH11 is shown with the residue numbers for secondary structure elements and the location of the catalytically essential residues E89 and E180 is indicated.

To study the structure function relationship of a new xylanase from the GH11 family the crystal structure of *N. haematococca* xylanase (NhGH11) was determined and refined to atomic resolution (1.0 Å). The overall 3D structure of NhGH11, consisting in total of 193 amino acids, is displayed in a single domain with β-jelly roll folding. The structure contains two β-sheets consisting of 14 β-strands (Fig. 1A). The smaller sheet A contains five strands forming a convex shaped outer layer, whereas the inner layer formed by sheet B is concave shaped and contains nine strands (Fig. 1B). All β-strands are antiparallel to each other except β8 and β11. Further, the structure contains one α-helix which is located between the loop connecting strands β12 and β13. Overall, the three-dimensional structure resembles a right hand with the fingers represented by two antiparallel β-sheets and a thumb loop located between strands β10 and β11. The inner antiparallel and highly twisted sheet creates a well-structured cylindrical cavity where xylan substrates bind and catalytic residues E89 and E180 are located. The overall fold is characteristic of the family 11 xylanases.

#### Sequence homology

A detailed comparison of GH11 sequences has been carried out for 82 sequences. Mature sequences of GH11 have been selected from Carbohydrate active enzyme database (http://www.cazy.org/GH11.html), including 47 sequences from fungi and 35 from bacteria. The results showed that the highest sequence homology is observed for the β-strands β5, β8, β10 and β11 also the C-terminal region, while the N-terminal region has less conserved amino acids. The three-dimensional structures of endo β-1,4 xylanases from family 11 are single-domain xylanases with overall homologous structures which can be superimposed with root mean square deviations (RMSD) of less than 1.5 Å for the Cα atoms. The catalytic residues E89 and E180 (numbering to NhGH11) and residues in the catalytic cleft of GH11 enzymes are highly conserved both in sequence and in space^21^.

**Figure 2:**
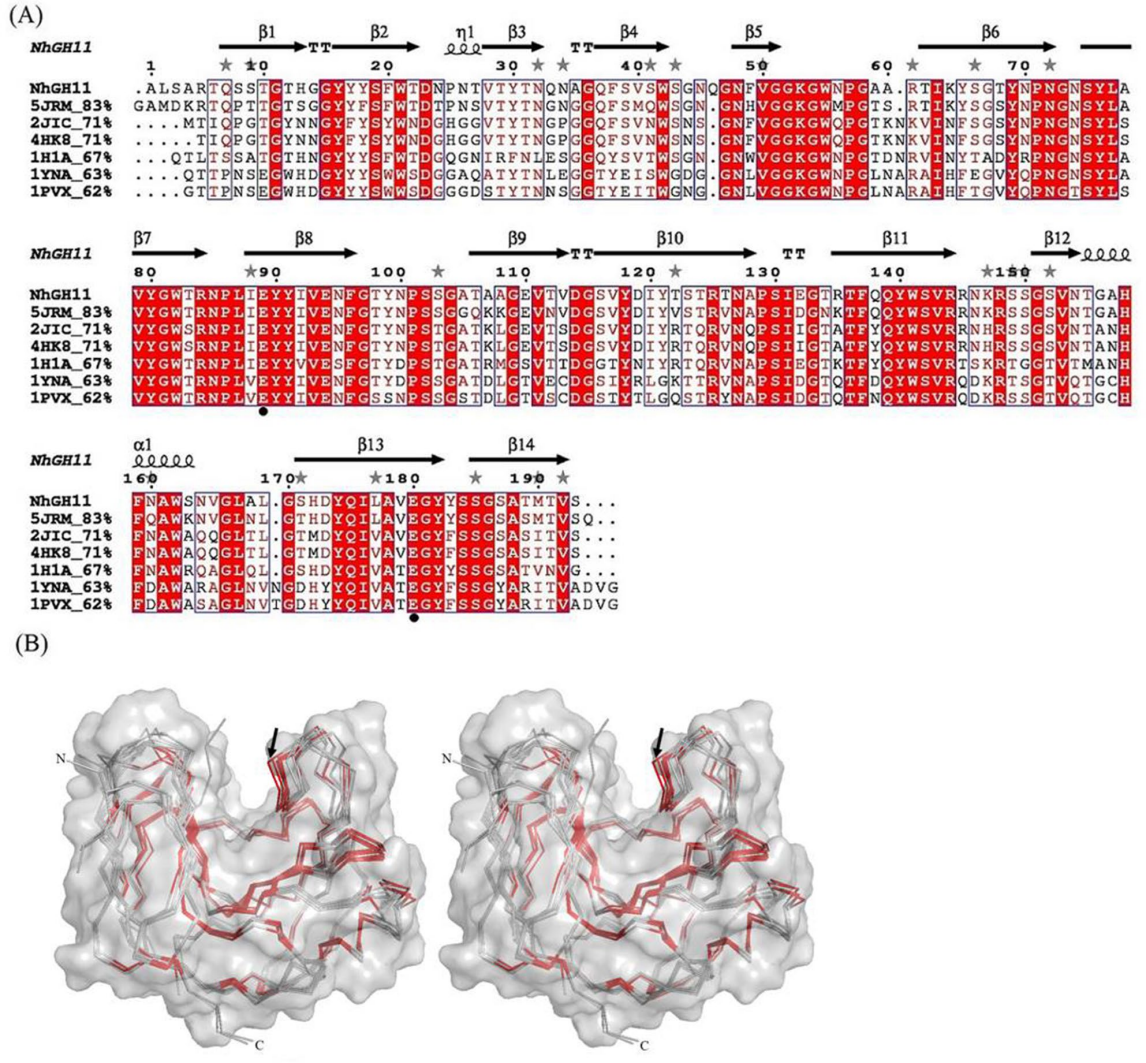
(**A**) Sequence alignment and comparison with homologous GH11 enzymes. Secondary structure regions are indicated along with gray stars indicating residues with alternative side chain conformations in the NhGH11 structure. Sequence alignment was done applying ESPript3 (http://espript.ibcp.fr/ESPript/ESPript). Residues in red color are identical and residues in pink color show similarity among aligned sequences. Catalytic residues E89 and E180 are indicated with filled circles. For the structure with pdb code 4HK8 the second catalytic E180 was mutated to Q180 for ligand binding studies (**B**) Stereo view of the surface representation with under laid Cα tracing. NhGH11 superimposed with homologue structures (pdb codes: 5JRM, 2JIC, 4HK8, 1H1A, 1YNA and 1PVX) from *F. oxysporum*, *T. longibrachiatum*, *H. jecorina*, *C. thermophilum*, *T. lanuginosus* and *B. spectabilis* respectively. Backbone in red color indicates regions with sequence identity for all structures, the highly conserved thumb region is indicated by black arrows.

We aligned the amino acid sequence of NhGH11 to the closest homologous sequences from *F. oxysporum*, *T. longibrachiatum*, *T. reesei*, *C. thermophilum*, *T. lanuginosus* and *B. spectabilis*, showing 83%, 71%, 71%, 67%, 63% and 62% sequence identity respectively (Fig. 2A). Superposition of the NhGH11 structure to respective homologous structures with pdb codes: 5JRM, 2JIC, 4HK8, 1H1A, 1YNA and 1PVX showed corresponding RMSD values of 0.48, 0.47, 0.55, 0.64, 0.63 and 0.58 Å for Cα atoms respectively. The RMSD values of superimposed structures correlates well with the sequence similarities, highlighted and shown for spatially conserved regions (Fig. 2B). Approx. 60% of the secondary structure elements correspond to β-strands and ~ 5% to the single α-helix.

For NhGH11 the highest sequence homology has been observed in β-strands β6, β7 β8 and β11. The thumb like loop between β10 and β11 strands showed highly conserved amino acids, P129, S130, and I131, important for enzyme activity and release of product after catalysis. The deletion of these residues drastically changed the active enzyme conformation to an inactive form^22^. Among aromatic residues several are highly conserved, as W42, W55, Y69, Y76, Y80, W82, Y90, Y91, F96, Y118, F137, W141, F158, W161, Y175 and Y182, while F21, W22 and Y66 (numbering to NhGH11) are less conserved regarding to the type of aromatic amino acid.

#### Active site

**Figure 3:**
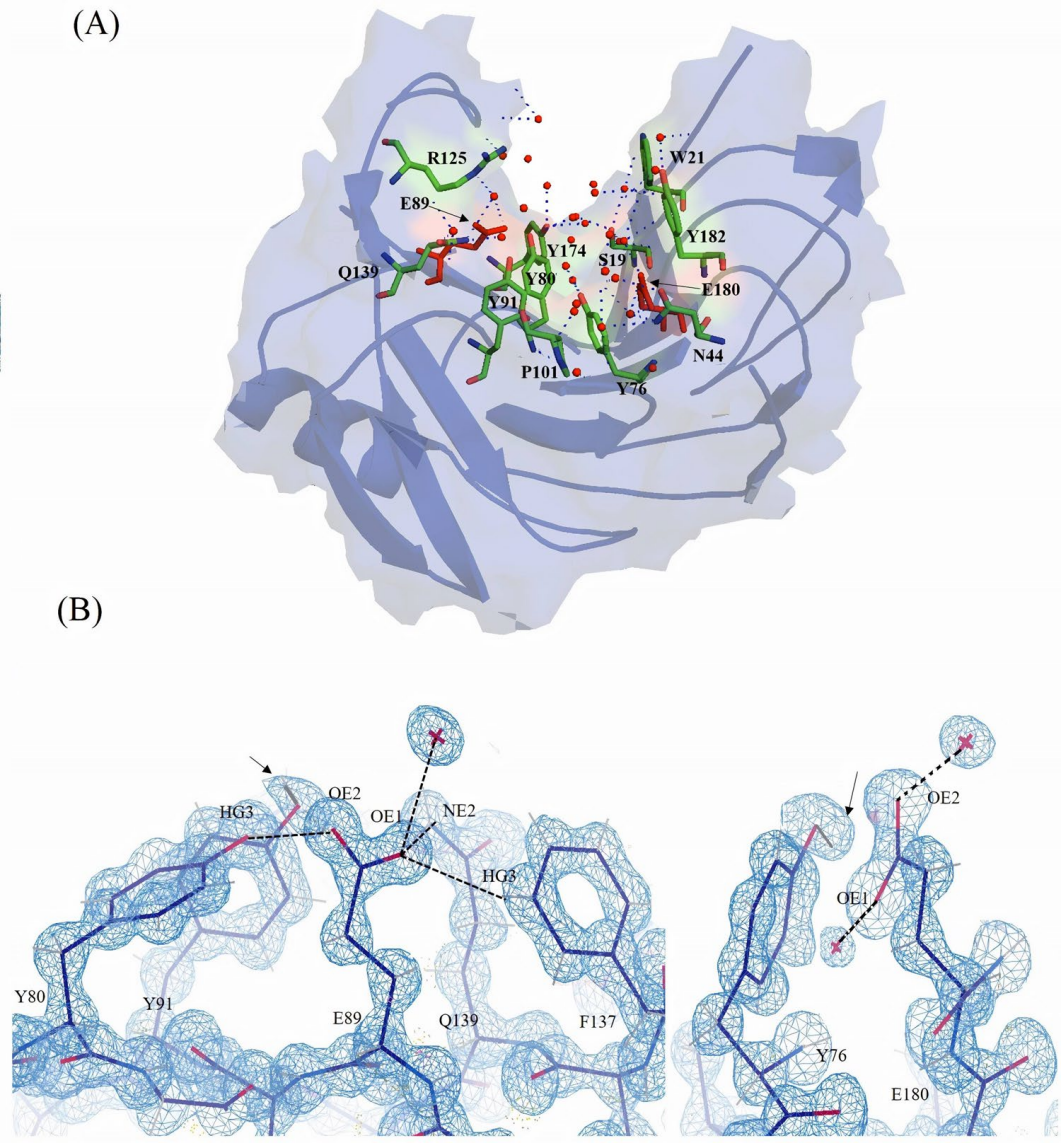
(**A**) Cartoon figure with residues highlighted in stick mode lining the active site cleft and forming hydrogen bonds with solvent molecules. (**B**) Catalytic residues E89 and E180 are forming hydrogen bonds with solvent molecules and surrounding residues. The positions of clearly visible hydrogen atoms in active site cleft are indicated by black arrows. 2Fo-Fc electron density maps are shown at 1.0σ level and all shown hydrogen bonds have distances of ~ 3.0 Å.

The catalytic residues E89 and E180 are located in the cleft with a side chain distance of ~ 10.5 Å. Overall the active site cavity is ~ 11 Å deep, ~ 4.5 Å wide and ~ 26 Å long (Fig. 3A). The active site cavity is surrounded by aromatic amino acids W21, Y76, Y80, Y91, Y174 and Y182. The overall structure of NhGH11 and active site matches with the xylanase from *T. reesei* (pdb code: 4HK8) with an RMSD value of 0.51 Å for Cα atoms. Inside the active site cavity W21, S19, R125, P101 and Y182 are reported to provide water mediated hydrogen bonds, while Y174, Y80, E80, Q139, Y91 and Y76 can provide direct hydrogen bonds to substrate, as reported for the structure 4HK8^15^. The assignment of active site residues and their roles in NhGH11 are proposed after superimposing the structure of the *T. reesei* enzyme-xylohexose complex. It can be expected that NhGH11 can process a similar substrate and the comparative analysis confirmed that the extended active site cleft of NhGH11 can also accommodate a linear xylan backbone. At one end of the active site cleft N74 is reported to be important to assign the pH-optimum of hydrolytic activity during catalysis^23,24^.

The details of hydrogen bonding and alternative conformations of twenty four amino acid side chains are visible in the high resolution structure of NhGH11. Inside the active site the carboxyl group of E89 (OE1) is hydrogen bonded to nitrogen (NE2) of Q139, hydrogen (HG3) of F137 and a water molecule (W49), while the second oxygen of the carboxyl group (OE2) is forming a hydrogen bond to Y80. The second catalytic residue E180 is forming hydrogen bonds to two water molecules via oxygen atoms OE1 and OE2 of carboxyl group, E180 strongly bends towards the main chain because the oxygen atom OE1 is forming a hydrogen bond with the main chain nitrogen of S75 (Fig. 3B). The high resolution structure allowed the analysis of hydrogen bonds throughout the active site cleft. The positions of hydrogen atoms provide information about the complex intramolecular hydrogen bond network.

#### Elucidation of thermostability parameters

**Figure 4:**
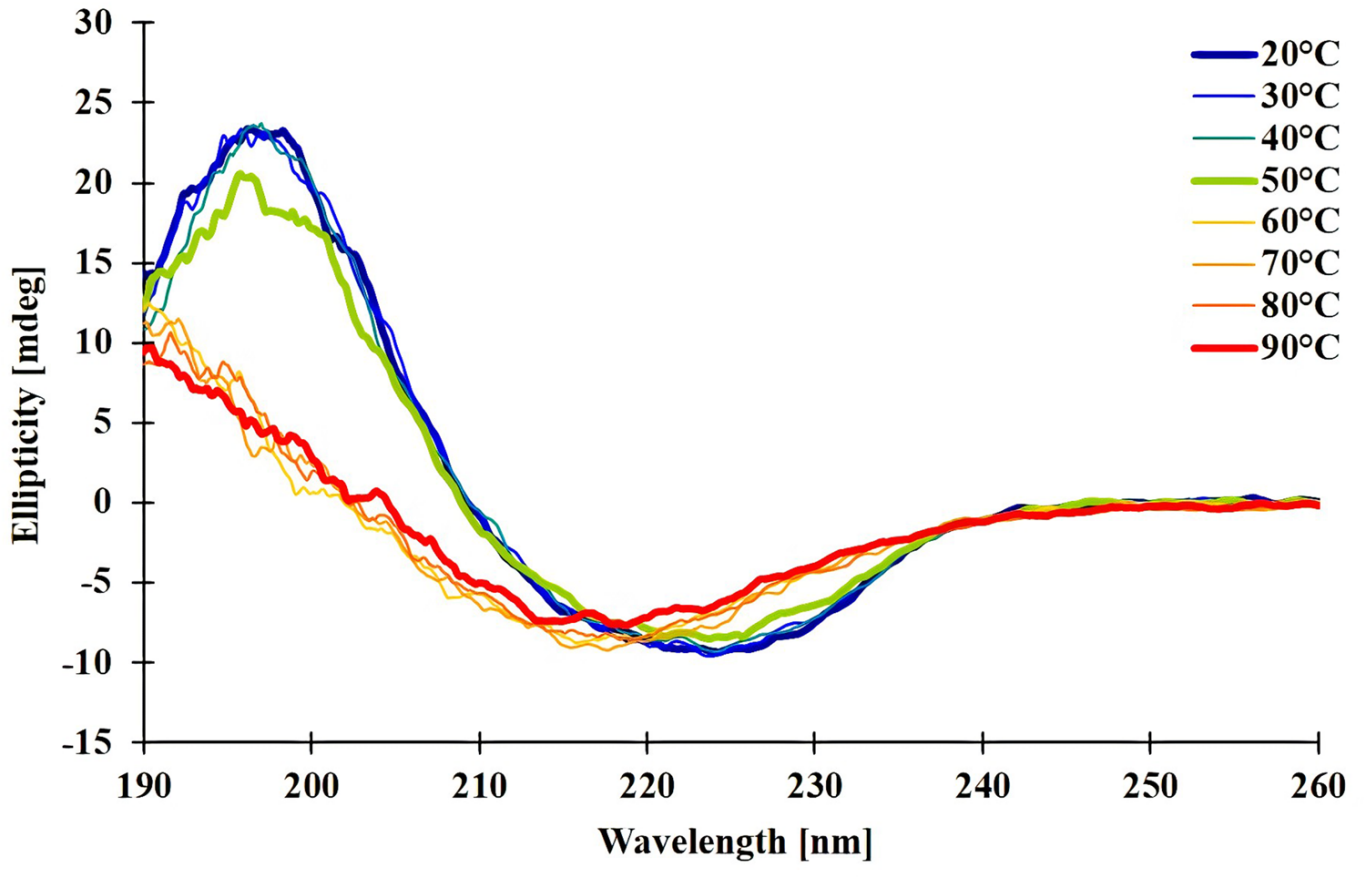
Circular Dichroism (CD) spectra at different temperatures recorded in the range of + 20 to + 90 °C, measurements were done with 10 °C intervals.

The thermostability was analyzed by following the thermal unfolding via Circular Dichroism (CD). CD data indicated that the secondary structure of NhGH11 is stable up to 40 °C. At temperatures higher than 50 °C the α-helix denatured first, while β-sheets remain partially stable up to 90 °C (Fig. 4).

**Figure 5:**
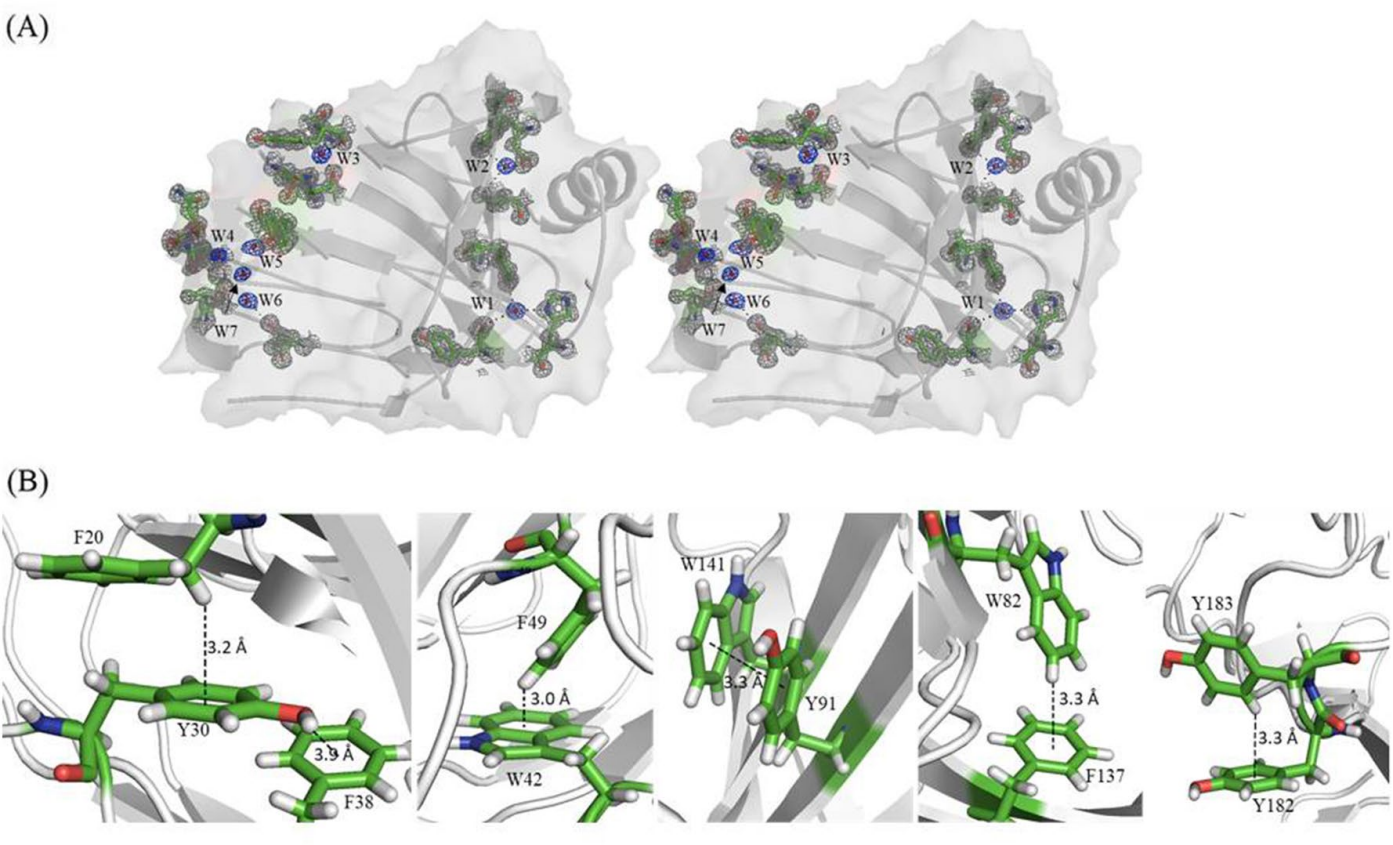
(**A**) Stereo figure showing buried water molecules with electron density within in the cartoon surface representation. 2Fo-Fc electron density maps for water molecules and surrounding residues are shown at 1.0σ level. (**B**) Selected intramolecular aromatic-aromatic ($\uppi{\cdots}\uppi$) interactions and ${\text{C-H}}{\cdots} \uppi$ interactions. Interactions were considered if the distance between the interacting proton and centroid of the phenyl group is less than 3.5 Å.

Extensive studies have been performed to identify structural features of xylanases related to thermostability^25–29^. Structural parameters supporting thermostability of xylanases include the number of disulfide bonds, hydrogen bonds and aromatic side chains forming stacking pairs and buried water molecules^30^. A calculation of solvent accessibility shows that NhGH11 contains seven buried water molecules (Fig. 5A). Aromatic side chain interactions forming π^…^π and C–H^…^π interactions include F20-Y30-F38, W42-F49, Y91-W141, W82-F137 and Y182-Y183 also contributing to the overall thermostability of NhGH11 (Fig. 5B). Interactions and sequence factors contributing to the thermostability of NhGH11 are compared with thermophilic GH11 xylanases (Table 1).

**Table 1:** Thermostability factors.

Xylanase	NhGH11	*D. thermo-philum*	*C. thermo-philum*	*T. lanugi-nosus*	*P. varioti bainier*	*T. reesei*
PDB code	6Y0H	1F5J	1H1A	1YNA	1PVX	1XYN
Number of residues	189	199	191	194	194	178
Optimum temperature (°C)	45	75^31^	60^32^	65^33^	65^34^	50^35^
Ion pairs	**10**	10	**18**	18	13	9
Hydrogen bonds (main-chain-main-chain)	163	163	163	163	157	152
Hydrogen bonds (main-chain-side-chain)	77	71	70	67	69	55
Hydrogen bonds (side-chain-side-chain)	**61**	**89**	71	81	77	68
Aromatic interactions	11	15	12	20	19	13
Hydrophobic interactions	**140**	**160**	144	127	115	135
No of Pi-Pi interactions	6	7	6	6	6	6
Arg/Lys ratio	**2.6**	2.5	**3.3**	2.6	3.0	3.0
Thr/Ser ratio	**0.87**	1.40	**1.41**	1.38	0.95	0.78
Asn + Gln	**26**	28	24	20	**21**	29
Cys residues	0	3	0	2	2	0
Aromatic residues	32	29	30	30	30	22
Buried water molecules	7	7	6	6	7	8

The highest differences between thermophilic GH11 enzymes and mesophilic NhGH11 are indicated bold.

For example, all thermophilic xylanases have more side-chain-side-chain hydrogen bonds compared to NhGH11 and thermophilic xylanases from *C. thermophilum, T. lanuginosus* and *P. varioti bainier* have more ion pairs as compared to NhGH11.

The comparison of twelve xylanases in relation to their thermostability parameters indicated some minor modifications such as higher Arg/Lys and Thr/Ser ratio, or lower number of Asn and Gln residues can enhance the thermostability^35^. Most of the thermophilic xylanases under comparison have a higher number of Arg/Lys and Thr/Ser ratio as compared to NhGH11*.* Thermophilic xylanases from *P. varioti bainier*, *T. lanuginosus* and *C. thermophilum* have lower number of Asn and Gln residues as compared to NhGH11. Another structural feature supporting thermostability of *D. thermophilum* xylanase is a 3_10_-helix located in the middle of the structure and also stabilizing the C-terminus, not present in NhGH11. This 3_10_-helix forms 34 hydrogen bonds^36^, compared to 14 hydrogen bonds formed by the corresponding region in the mesophilic NhGH11. Based on comparison of structural parameters supporting thermostability of xylanases, the mesophilic character of NhGH11 is obvious.

### Biochemical characterization

#### Effect of temperature and pH

The effect of temperature and pH on the activity of NhGH11 was investigated to identify the optimum conditions for enzyme catalysis. The effect of temperature was studied from 30–80 °C applying 5 °C intervals. The enzyme showed activities scaled to 92.9 ± 1.8%, 100.7 ± 3.8% and 84.8 ± 2.7% at 40 °C, 45 °C, and 50 °C respectively. And, NhGH11 showed 76.4 ± 0.9%, 100.0 ± 3.5% and 66 ± 0.55 ± 1.26% relative activities at pH 5, 6 and 7 respectively. Relative activities at different pH and temperatures were calculated by considering the activity to be 100% at 45 °C and pH 6.0, as shown in supplementary material (S2 A and B). The optimum pH of GH11 xylanases depends on the amino acids located in the vicinity close to the second catalytic glutamate, e.g. xylanases with a rather alkaline pH optimum (pH_opt_ > 5) have asparagine, whereas acidophilic ones have aspartate (pH_opt_ < 5) in the vicinity close to the second catalytic glutamate^24^. For NhGH11 asparagine N74 is vicinal to catalytic E180, which indicates that NhGH11 is an alkaline xylanase, which correlates to the observed pH optimum.

The temperature dependent stability of NhGH11 was analyzed at different temperatures (40 °C, 45 °C and 50 °C) for a period of 8 h as shown in supplementary material (S2C). Activity at optimum conditions was considered as 100%. The enzyme showed 80% activity at 40 °C and 45 °C after a period of 4 h and then decreased to approx. 50% and 45% after 8 h. At 50 °C the enzyme lost 50% of its activity after 4 h. The optimum temperature for most of the xylanases is in a range between 35 and 85 °C, the temperature preference categorized the xylanases as mesophilic or thermophilic enzymes^24^. Based on these observations we can categorize NhGH11 as a mesophilic xylanase that can be utilized for industrial applications operating in a temperature range of 40–50 °C.

#### Kinetic parameters and substrate selection

**Figure 6:**
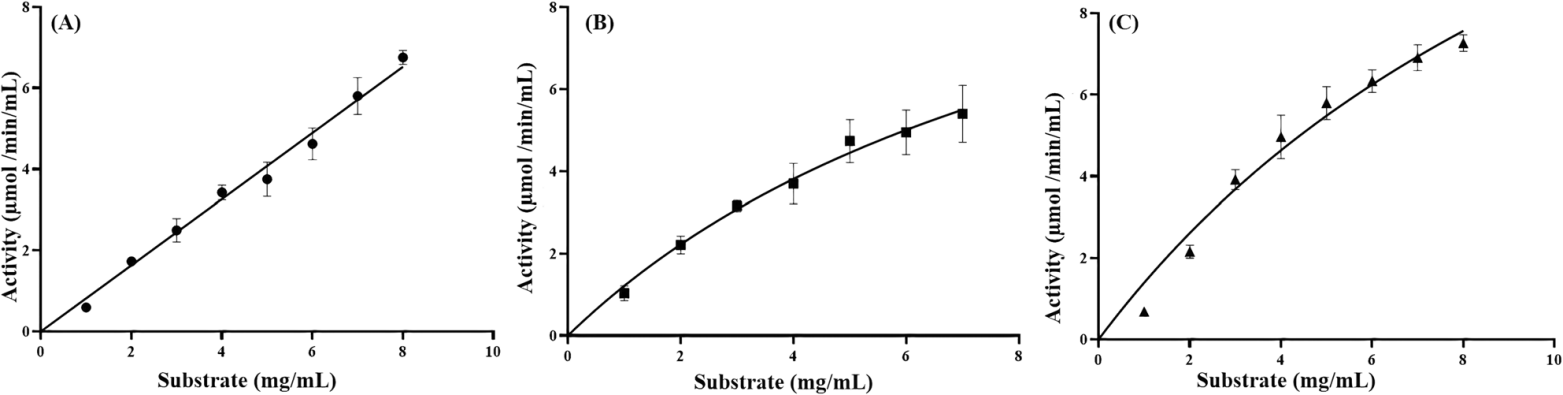
Kinetics with different substrates (**A**) Activity with beechwood xylan (**B**) Activity with xylan polysaccharide (**C**) Activity with wheat arabinoxylan.

Kinetic parameters applying different substrates were analyzed (Fig. 6). NhGH11 was found to be active towards beechwood xylan, xylan polysaccharide and wheat arabinoxylan but not towards azo-xyloglucan and mannan. The specificity constant, k_cat_/K_m_ (mg^−1^ ml min^−1^), applying beechwood xylan as substrate was higher than for wheat arabinoxylan and xylan polysaccharides, while the turnover number, k_cat_ (min^−1^), for wheat arabinoxylan was higher than for beechwood xylan and xylan polysaccharides (Table 2). Endo β-1,4 xylanases from *P. oxalicum* and *Bis pora* also showed the highest specific activity applying beechwood xylan as substrate, followed by oat spelt xylan and birchwood xylan^37^. Mostly, GH11 xylanases are known to have higher specific activity for xylans in comparison to GH10 xylanases, which are known to hydrolyze a broad range of different substrates^38^. NhGH11 showed the lowest K_m_ for beechwood xylan, similar activity for xylanase from *A. niger*, indicating a higher affinity towards beechwood xylan^39^.

**Table 2:** Comparison of kinetic values utilizing distinct substrates.

Substrate	K_m_ (mg/ml)	V_max_ (µM/min)	k_cat_ (min^−1^)	k_cat_/K_m_ (mg^−1^ ml min^−1^)
Beechwood xylan	8.05 ± 2.08	14.07 ± 2.58	1730.63 ± 318.06	214.98 ± 68.17
Xylan polysaccharide	10.07 ± 2.28	13.41 ± 2.02	1648.22 ± 249.29	163.68 ± 44.67
Wheat arabinoxylan	12.96 ± 4.1	19.67 ± 4.2	2410.82 ± 517.46	200.90 ± 81.06

#### Identification of products

The hydrolytic products released by NhGH11 were analyzed by matrix-assisted laser desorption ionization-time of flight mass spectrometry (MALDI-TOF-MS). The products were identified applying positive ion mode mass spectra for smaller oligosaccharide products, as sodium adduct ions (M + Na^+^), for which the molecular weight was calculated and summarized in supplementary material (S3). The indicated spectra showed peaks corresponding to 100–800 Da. In the selected range, four peaks were identified corresponding to xylooligosaccharides sodium adducts, i.e. xylobiose (304 Da), xylotriose (436 Da), xylotetrose (568 Da) and xylopentose (700 Da), indicating that NhGH11 can cleave xylan into oligosaccharides of different lengths and monomers, as xylose (X), xylobiose (X2), xylotriose (X3) and xylotetrose (X4). The proposed mechanism may include the cleavage of X5 into X4 and X, X4 into X3 and X2, X3 into X2 and X. The peak corresponding to a xylose monomer was not well identified in the MALDI-TOF spectra, but the presence of X4 indicated that X should be present. A difference in the substrate binding site of xylanases can typically change the ratio of different products and cleavage specificity depends on the GH family of xylanase^40^. The xylanase from *T. turnerae*^41^ belongs to GH8 family cannot cleave xylooligosaccharides with less than X4, while thexylanase from *P. oxalicum*^37^ belongs to GH11 family can efficiently degrade X4 and longer xylan oligosaccharides. The catalytic activity of NhGH11 producing smaller xylooligosaccharides can be considered as an important and valuable enzymatic property for a number of industrial applications, as resulting xylooligosaccharides can be used as food ingredients and can act as a prebiotic to maintain the intestinal function^42^. Furthermore, such xylooligosaccharides have been categorized as nutraceuticals because of their potential application to prevent atherosclerosis, anti-inflammatory, anti-hyperlipidemic and anti-cancer activities. Besides these biological functions xylooligosaccharides have also phytopharmaceutical and feed applications^43–45^.

In this context the β-1,4 xylanase activity of NhGH11 can be considered for a number of industrial applications to produce distinct hydrolytic products.

#### Effect of metal ions and chemical reagents

Xylanases are applied today in different industrial processes and since metal ions and several chemical agents are usually present in industrial process environment^46^, we analyzed the effect of selected metal ions and chemical reagents in context of the activity of NhGH11. Details are summarized in supplementary material (S4).

Na^+^ and K^+^ ions did not affect the enzyme activity of NhGH11, while the activity gradually decreased from 82.5 ± 3.8% to 52.2 ± 3.6% in presence of Ca^2+^, Co^2+^, Mg^2+^, Zn^2+^ and Mn^2+^. With Fe^2+^ and Cu^2+^ a more severe reduction of enzyme activity was observed namely to 25.6 ± 4.3% and 15.6 ± 2.6% respectively (S4A). The sensitivity of xylanases towards metal ions is not predictable among members of the GH11 family. For example Mn^2+^ and Zn^2+^ enhanced the activity of *A. terreus* xylanase^47^ while Mn^2+^ and some other divalent ions slightly reduced the activity of *P. oxalicum* xylanase. For *P. oxalicum* xylanase, metal ions activated the enzyme at lower concentrations but strongly deactivated it at higher concentrations^37^.

The effect of selected chemical reagents on the activity of NhGH11 showed that sodium azide, tween-80 and triton X-100 reduce the activity to 91.0 ± 2.6%, 80.1 ± 5.0%, and 70.2 ± 4.9% respectively (S4 B). NhGH11 showed a significant decrease in activity in the presence of EDTA and SDS. The decreased enzyme activity in the presence of EDTA, as a metal chelating agent, indicated that at low concentrations some divalent metal ions are supporting the activity of NhGH11.

### Conclusions

The three-dimensional structure of NhGH11 has the typical fold of family 11 xylanases with a single catalytic domain and conserved active site residues. The overall structure contains two β-sheets and one α-helix forming a complex network of hydrogen bonds that substantially supports the stability of NhGH11. The crystal structure refined with anisotropic B-factors to 1.0 Å resolution allowed to investigate hydrogen atom positions involved in main-chain interactions, side-chain interactions and even alternative conformations of amino acid side chains. High resolution data also allowed to analyze hydrogen bonds in the active site, as well as alternative conformations of solvent molecules. The active site of NhGH11 is compatible to the xylanase from *T. reesei*, PDB code: 4HK8 enzyme-xylohexose complex and as a result NhGH11can be expected to process a similar substrate.

In summary, our structural and biochemical investigations allowed to understand the molecular structure and activity parameters, such as optimum temperature, pH and thermostability. We could also identify that NhGH11 has relative low sensitivity towards most common metal ions and chemical reagents present in potential industrial applications. NhGH11 is a mesophilic xylanase that can catalyze the efficient breakdown of xylan-based substrates at an optimum temperature of 45 °C and a pH of 6.0, and due to broad applications of its hydrolytic products NhGH11 can be considered for a wide range of corresponding industrial processes performed between 40 and 50 °C.

## Material and methods

### Gene expression and purification

The sequence of NhGH11 (GenBank ID: GG698899.1, Uniprot ID: C7YSL3) was analyzed applying the SignalP 4.1 server to identify the attached signal peptide and its cleavage site (http://www.cbs.dtu.dk/services/SignalP-4.1/). The surface entropy reduction prediction server (http://services.mbi.ucla.edu/SER/) was utilized to identify high entropy residues^48^ such as K4, K107, K108 and K168, which were subsequently mutated to alanine to support crystallization experiments. The corresponding gene was synthesized and supplied by Biocat (Heidelberg, Germany) ligated in the plasmid (pMAL-c5x) for transformation in order to express amino acids 30-222 using *E. coli* strain BL21 (DE3). Purification was done applying the maltose-binding protein (MBP) as a fusion partner by using a standard affinity chromatography protocol^49^. The purified enzyme was incubated for 12 h at 4 °C with 1 mg of TEV protease per 10 mg of a fusion protein. The TEV protease was removed by Ni–NTA affinity resin after MBP tag cleavage. The flow-through sample was concentrated to 5 mL and loaded onto a Hi Load 16/600 Superdex 75 gel filtration column (GE Healthcare) equilibrated with buffer containing 20 mM Tris–HCl pH 7.4 and 200 mM NaCl. Sample solutions were analyzed by SDS-PAGE to check their purity and integrity. Dynamic light scattering was applied to analyze the homogeneity of the enzyme prior to crystallization experiments.

### Crystallization, diffraction data collection and structure refinement

Initial crystals were obtained by the hanging-drop vapor diffusion method, equilibrating over mother liquor consisting of 1 M ammonium sulfate, 100 mM sodium citrate pH 5.5 with equal volumes of 12 mg/mL protein at + 20 °C. After 2 days inter-grown thin plate like crystals were obtained with dimension up to 200 µm. To obtain single crystals with larger volume micro-seeding was performed and uniformly shaped crystals with dimensions of ~ 100 µm were obtained. Prior to diffraction data collection, crystals were briefly soaked in cryo-protectant containing mother liquor and 20% glycerol. Diffraction data were collected at the EMBL beamline P13 (PETRA III, DESY, Germany) at 100 K, applying a X-ray wavelength of 0.97 Å. The collected data were processed applying the XDS package^50^. The structure was determined by molecular replacement using the homologous structure from *F. oxysporum* xylanase (PDB code: 5JRM), having 82% sequence homology. Refinement was done applying PHENIX 1.8.4_1496. For 20 cycles of refinement default values for all parameters, including automated weighting between geometry and X-ray target functions were used and the correct amino acids were introduced in the electron densities. Further, refinement with isotopic B-values was performed and R_work_/R_free_, converged to 0.17/0.18. During following model building in total 247 solvent water molecules were introduced. Model building was done applying COOT^51^. No electron density for the N-terminal residues 1–4 was observed. For determining high resolution cutoff of the highest resolution shell (1.03–1.00 Å), the criteria I/σI ≈ 2.3 and CC_1/2_ > 50% was applied and all atoms, except hydrogens, were refined anisotropically^52^. Further, 14 additional cycles of anisotropic refinement were performed using all data and including the hydrogen atoms. Thirty one additional solvent molecules and alternate conformation of twenty four residues were introduced. All solvent molecules were introduced with appropriate occupancies and hydrogen bond distances. The quality of the structure after anisotropic refinement was analyzed applying REDO^53^ and the PDB validation server^54^. The obtained average atomic B-value was 10.78 Å^2^ and individual B-values range from 5.70–32 Å^2^. During anisotropic B-factor refinement the average errors for chemical bonds and angles between main-chain atoms and side chains were monitored. P57 and P86 adopted a cis conformation, as shown in supplementary material (S5). The refinement converged with ideal stereo chemical parameters and R_work_/R_free_ 0.14/0.15 (Table 3). The coordinates are deposited in protein data bank (www.rcsb.org) with pdb code 6Y0H.

**Table 3:** Data-collection and refinement statistics.

Data collection	
X-ray source	P13 beamline PETRA III, DESY
Detector	Pilatus
Space group	C 2 (No. 5)
Cell dimensions	
a, b, c (Å)	79.45, 38.50, 53.59
α, β, γ (º)	90.00, 91.43, 90.00
Wavelength (Å)	0.97
Resolution range (Å)	34.6–1.0 (1.03–1.0)
Total number of reflections	258,391 (33,253)
Redundancy	3.1 (2.6)
Wilson B-factor (Å^2^)	6.5
R_meas_	0.053 (0.49)
R_merge_	0.045 (0.47)
CC_1/2_	0.999 (0.857)
I/σI	15.57 (2.33)
Completeness (%)	93.12 (78.74)
Refinement	
Reflections used	82,543 (6956)
Reflection used for R_free_	1970 (174)
R_work_	0.14 (0.24)
R_free_	0.15 (0.25)
No. atoms	1863
Protein	1580
Ligand/ion	/
Water	278
Average B-factor (Å^2^)	10.78
For macromolecule	8.66
For water	22.61
R.m.s deviations	
Bond lengths (Å)	0.008
Bond angles (º)	1.37
Ramachandran	
Favored (%)	97.85
Allowed (%)	2.15
Outliers (%)	0.00
PDB code	6Y0H

*Data in highest resolution shells are shown in parenthesis.

### Biochemical characterization

#### Effect of temperature and pH

The optimum temperature, pH and temperature dependent stability of NhGH11 was investigated applying a DNS assay^55^, utilizing β-1,4 beechwood xylan as substrate (Megazyme, Australia). Purified NhGH11 (0.16 mg/mL) was incubated with 5 mg/ml substrate for all experiments. To identify the optimum temperature, the reaction mixture was incubated at different temperatures (30–80 °C) in 50 mM sodium citrate buffer at pH 6.0. For identifying the optimum pH, the reaction mixture was incubated in a wide range of 100 mM McIlvaine buffers (pH 2–11). For investigating the temperature dependent stability the enzyme was incubated in 50 mM sodium citrate pH 6.0 at 40 °C, 45 °C, and 50 °C for 8 h prior to measuring the activity. All reactions were completed by addition of 3,5-dinitrosalicylic acid (DNS) and the absorbance of each sample was measured at 510 nm, data were represented as the mean of triplicates with the respective standard deviation.

Circular dichroism (CD) spectroscopy^56^ was used to analyze the secondary structure and thermostability of the NhGH11 using a JASCO J-815 CD spectrometer (Jasco, UK). NhGH11 was diluted to 0.12 mg/mL in 5 mM NaF to record the melting curve at 10 nm/min scanning speed within a temperature range of 20–90 °C, applying an increment of 1 °C/min. The measurement was done in a quartz cuvette (1 mm path length) and applying a wavelength rangeof 190 to 260 nm. The ellipticity was plotted against the wavelength and temperature using the software J-815 Spectra manager (Jasco, UK).

#### Kinetic parameters and substrate selection

The catalytic activity of NhGH11 was analyzed towards beechwood xylan (β-1,4 linked xylan with 8.7% of 4-O-methyl glucuronic acid and 5.7% other sugars, Sigma, USA), xylan polysaccharide (β-1,3- and β-1,4-linked D-xylose polymer, Elicityl, France), wheat arabinoxylan (β-1,4-linked xylan with overall 36% of arabinose, 51% xylose, 6.5% glucose and 4.4% of mannose sugars, Megazyme, Australia), azo-xyloglucan from tamarind (β-1,4 linked glucose with branches of galactose, xylose, arabinose, and other sugars, Megazyme, Australia) and mannan polysaccharide (β-1,4-linked mannose with 2% galactose and other sugars, Sigma, USA) applying a DNS assay^55^. Hydrolytic reactions with the enzyme (0.16 mg/mL) and substrates (0.05–8.0 mg/mL) were performed at 45 °C for 10 min in 50 mM sodium citrate buffer at pH 6.0 in 100 µl final volume then immediately mixed at a 1:1 volume ratio with DNS solution and heated to 90 °C for 10 min. The absorbance of each sample was measured at 510 nm and data were processed as the mean of triplicate with the respective standard deviation. The standard xylose curve was prepared. The Michaelis Menten constant and maximum velocity was calculated using the software OriginPro (OriginLab Northampton, MA, USA).

#### Identification of products

MALDI-TOF-MS was applied to determine the molecular masses of fragmented ions originating from a degraded xylopentose (5-xyl) after incubation with NhGH11 at the optimum temperature and pH condition determined before. The previously established reaction conditions were performed in the citrate buffer, however at lower ionic strength (5 mM) for 15 min. The reaction was terminated by heating to 90 °C for 10 min. The hydrolyzed mixture was centrifuged at 16,000 ⨯ *g* for 30 min to remove the precipitated enzyme. MALDI-TOF–MS measurements were performed using an ultrafleXtreme mass spectrometer (Bruker Daltonik, Bremen, Germany) equipped with a 2 kHz smart beam-II Laser and by using dihydroxyacetophenone matrix. Data were automatically acquired using Flex control 3.0 and Maldi Biotyper Automation Control 2.0 software (Bruker Daltonics GmbH, Bremen, Germany). The method of identification included the m/z from 100 to 800 Da. For each spectrum a maximum of 100 peaks was considered, and the obtained data were compared with the expected molecular weights of xylooligosaccharides products as sodium adducts.

#### Effect of metal ions and chemical reagents

The effect of metal ions and chemicals reagents was studied applying a DNS assay^55^. Stock solutions of 100 mM of the metal salts (NaCl, KCl, CoCl_2_, CaCl_2_, MgCl_2_, ZnSO_4_, MnCl_2_, FeSO_4_, and CuCl_2_) were prepared and further diluted with 50 mM sodium acetate buffer pH 6.0 to 10 mM working concentration. Stock solutions of chemical reagents (tween-80, triton-100, β-mercaptoethanol, EDTA, SDS) were prepared in 50 mM sodium acetate buffer to 2% working concentrations, except for sodium azide (0.2%).

## Supplementary information

Supplementary file1
