# Supplementary material for: High-resolution crystal structure and biochemical characterization of a GH11 endoxylanase from Nectria haematococca
Source: Sci Rep. 2020 Sep 24;10:15658. doi: 10.1038/s41598-020-72644-w (PMC7519127; doi:10.1038/s41598-020-72644-w)
Supplement: Supplementary file 1 — Supplementary file1 [file 41598_2020_72644_MOESM1_ESM.pdf]

Supplimentary material

High-resolution crystal structure and biochemical characterization of a GH11 endoxylanase from *Nectria haematococca*

Hina Andaleeb,[1,2] Najeeb Ullah,[1,2], Sven Falke[1], Markus Perbandt,[1]

Hévila Brognaro[1] and Christian Betzel[1,3*]

[1] Institute of Biochemistry and Molecular Biology, Laboratory for Structural Biology of Infection and Inflammation, University of Hamburg, c/o DESY, Build. 22a. Notkestr. 85, 22603 Hamburg, Germany

[2] Department of Biochemistry, Bahauddin Zakariya University, Multan-60800, Punjab, Pakistan

[3] The Hamburg Centre for Ultrafast Imaging (CUI), Luruper Chaussee 149, 22761 Hamburg, Germany

*Correspondence: C.B: Christian.Betzel@uni-hamburg.de

Phone: +49 (40) 8998 - 4744

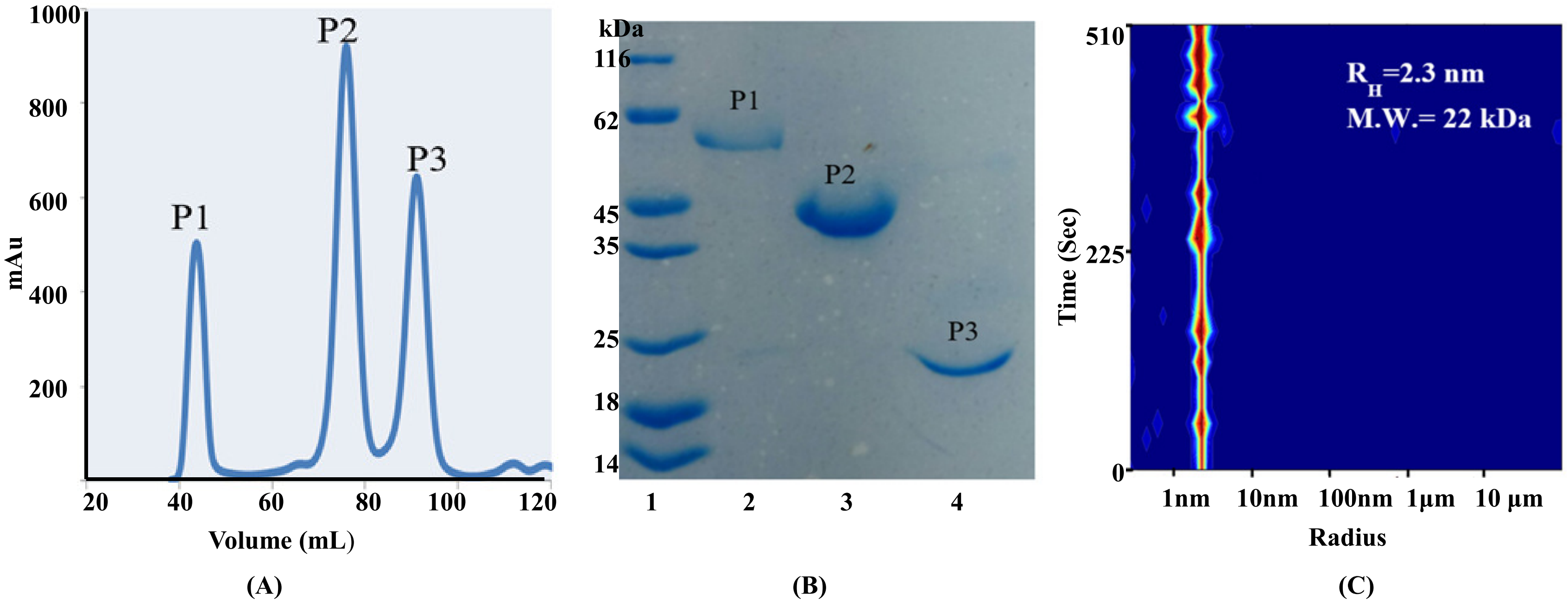

S1. (A) Size exclusion chromatography profile P1: NhGH11 with MBP, P2: MBP after cleavage, P3: purified NhGH11 after size exclusion chromatography. **(B)** SDS PAGE of size exclusion chromatography, lane 1: molecular marker, lane 2: NhGH11 with MBP (60kDa), lane 3: MBP after cleavage, approx. 40kDa, lane 4: NhGH11 after cleavage from MBP, approx. 22 kDa. **(C)** Dynamic Light Scattering (DLS) of NhGH11 (10 mg/ml), showing a monodisperse protein solution, radius R_H= 2.3 nm ± 0.03, which corresponds to approx. MW 22 kDa molecular weight.

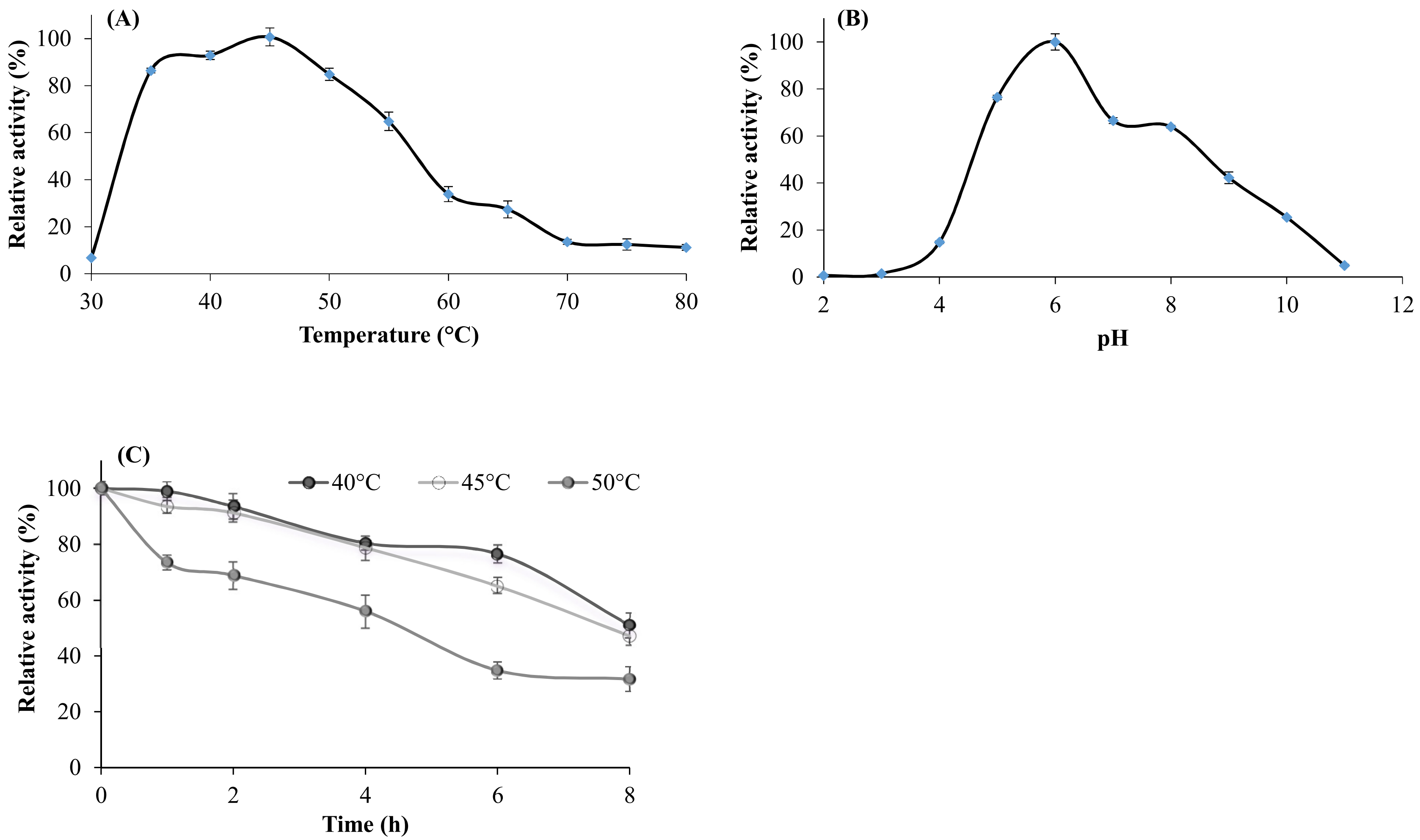

S2 . (A) The optimum temperature for enzymatic activity was determined by measuring the activity at different temperatures using 1 % xylan beechwood as substrate. **(B)** The optimum pH was determined in 100 mM Mcilvaine buffers (pH 2-11) using 1 % xylan beechwood as substrate. **(C)** The temperature dependent stability and activity was evaluated following the relative activity incubating the enzyme at 40ºC, 45ºC and 50ºC for 8 hours. The data points represent mean values of three replicates and bars indicate the corresponding standard deviation.

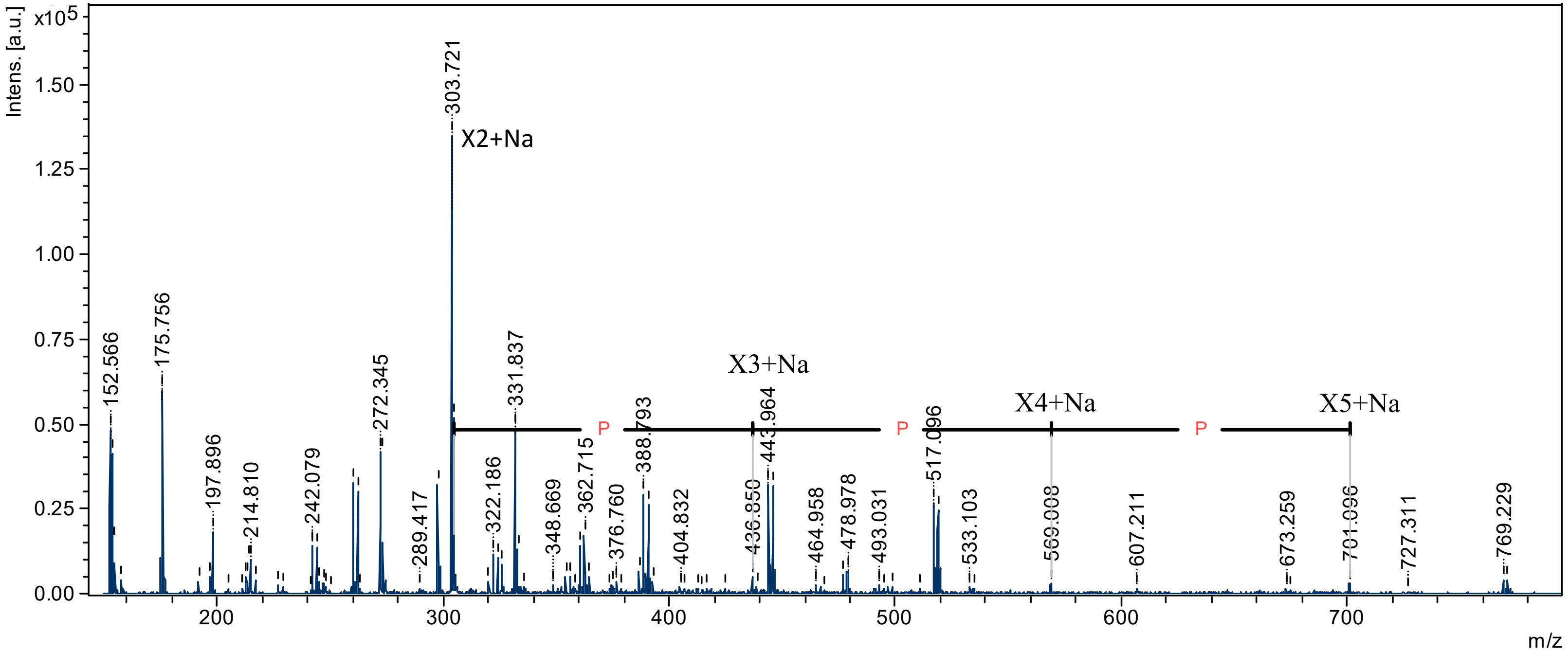

S3. Identification of catalysis products. MALDI-TOF MS spectrum of products (X2, X3, X4) formed by cleavage of the substrate (X5), measured by using 2,5-dihydroxybenzoic acid matrix, spectra represent the xylooligosaccharides adducts with sodium ions.

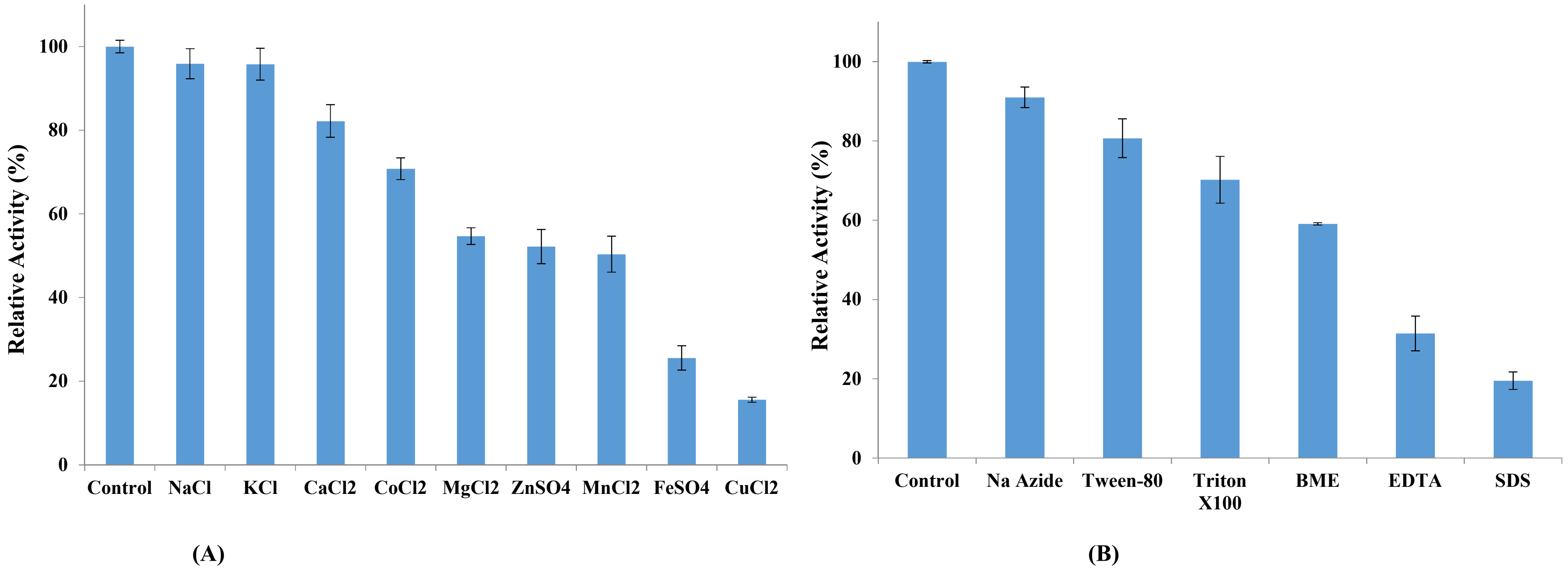

S4. (A) Relative activity (%) in presence of 10 mM metal ions. **(B)** Relative activity (%) in the presence of 2 % solutions of chemical reagents and 0.2 % solution of sodium azide. The activity of the purified enzyme at 45°C and pH 6.0 was assigned as 100% activity. The data represent a mean value of three replicates and bars indicate the standard deviation of the three replicates.

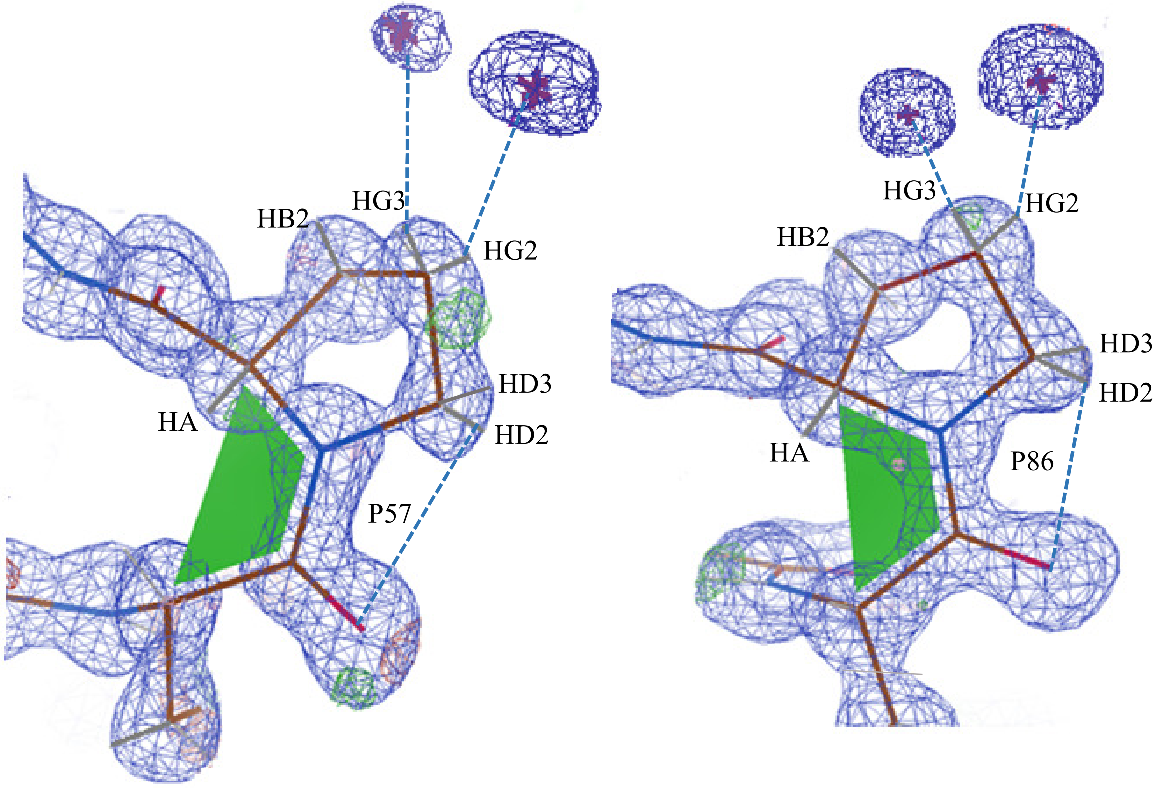

S5. Unconventional backbone conformations observed during refinement. P57 and P86 adopt cis conformations. H-bonds are indicated by dotted lines. 2Fo-Fc electron density maps are shown at 1.0σ level. The electron densities were calculated after anisotropic B-factor refinement.
